# Arbor-TVB: A Novel Multi-Scale Co-Simulation Framework with a Case Study on Neural-Level Seizure Generation and Whole-Brain Propagation

**Published:** 2025-05-22

**Authors:** Thorsten Hater, Juliette Courson, Han Lu, Sandra Diaz-Pier, Thanos Manos

**Affiliations:** 1 Simulation and Data Lab Neuroscience, Jülich Supercomputing Centre (JSC), Institute for Advanced Simulation, Forschungszentrum Jülich GmbH, Jülich, Germany; 2 ETIS Lab, ENSEA, CNRS, UMR8051, CY Cergy-Paris University, Cergy, France; 3 Department of Computer Science, University of Warwick, Coventry, UK

**Keywords:** Arbor, The Virtual Brain, Multi-scale Neural Models, Seizures, Mouse Brain Connectome

## Abstract

Computational neuroscience has traditionally focused on isolated scales, limiting understanding of brain function across multiple levels. While microscopic models capture biophysical details of neurons, macroscopic models describe large-scale network dynamics. Integrating these scales, however, remains a significant challenge. In this study, we present a novel co-simulation framework that bridges these levels by integrating the neural simulator Arbor with The Virtual Brain (TVB) platform. Arbor enables detailed simulations from single-compartment neurons to populations of such cell, while TVB models whole-brain dynamics based on anatomical features and the mean neural activity of a brain region. By linking these simulators for the first time, we provide an example of how to model and investigate the onset of seizures in specific areas and their propagation to the full brain. This framework employs an MPI intercommunicator for real-time bidirectional interaction, translating between discrete spikes from Arbor and continuous TVB activity. The novel Arbor-TVB co-simulator allows replacement of TVB nodes with biologically realistic neuron populations, offering insights into seizure propagation and potential intervention strategies. The integration of Arbor and TVB marks a significant advancement in multi-scale modeling, providing a comprehensive computational framework for studying neural disorders and optimizing treatments.

## INTRODUCTION

The human brain consists of billions of neurons and an equally vast population of non-neuronal cells, intricately organized into layers and regions (Herculano-Houzel, 2009, 2012). Each neuron operates as a highly sophisticated biochemical machinery (Augustine, Santamaria, & Tanaka, 2003; Darnell, 2013; Lu, Garg, Lenz, & Vlachos, 2025; West, Griffith, & Greenberg, 2002), coordinating signal transmission within an extensive network in health (Barral, Wang, & Reyes, 2019; Dicks, 2022; Reyes, 2003) and disease [see e.g., Tetzlaff et al. (2025)]. Ever since the Hodgkin-Huxley model was introduced to describe membrane potential dynamics (Hodgkin & Huxley, 1952c), computational neuroscience has played a pivotal role in enhancing our understanding of brain function. Yet, due to the immense complexity of the brain and computational constraints, most modeling studies focus on a single scale simulator or rely on standalone simulation codes.

Simulations incorporating biophysical properties and neural morphology typically concentrate on individual neurons using simulators such as the NEURON simulator (Carnevale & Hines, 2006). At the microscopic level, studies have explored questions such as how the ion channel kinetics influence neural excitability [see e.g., Gurkiewicz, Korngreen, Waxman, and Lampert (2011); Suma, Sigg, Gallagher, Gonnella, and Carnevale (2024)], how proteins, enzymes, and calcium concentration are distributed among neighboring spines to impact plasticity [see e.g., Chater, Eggl, Goda, and Tchumatchenko (2024); Luboeinski and Tetzlaff (2021)], and how signal propagation along axonal fibers relates to neuropathic pain [see e.g., Tigerholm et al. (2015, 2014)]. Some studies examine how neural morphology—such as dendritic tree growth [see e.g., Yasumatsu, Matsuzaki, Miyazaki, Noguchi, and Kasai (2008)] and morphology-dependent plastic interactions [see e.g., Hananeia et al. (2024)]—affects function. These studies, while often limited to small patches of the neural membrane, a few dendritic segments, or a small local network, provide valuable approximations of broader neural phenomena.

At the mesoscopic level, researchers simplify neuronal representations using point leaky-integrate-and-fire neurons (based on simulators such as NEST (Gewaltig & Diesmann, 2007) or Brian/Brian2 (Stimberg, Brette, & Goodman, 2019)), enabling studies on larger networks without explicit neuronal morphology or with some degree of self-customized morphology, using e.g., NESTML (Linssen et al., 2024). This approach has advanced our understanding of neural heterogeneity (Demirtaş et al., 2019; Gast, Solla, & Kennedy, 2024; Nayebi et al., 2021), self-organization (Diaz-Pier, Naveau, Butz-Ostendorf, & Morrison, 2016; Miner & Triesch, 2016; Zheng, Dimitrakakis, & Triesch, 2013), neural capacity (Emina & Kropff, 2022), energy efficiency (Sacramento, Wichert, & van Rossum, 2015), and neural plasticity in disease and health (Lu, Diaz, Lenz, & Vlachos, 2024; Manos, Diaz-Pier, & Tass, 2021). Most microscopic and mesoscopic models remain theory-driven, using mathematical approximations to infer neural behavior rather than directly establishing model based on large datasets [see e.g., Popovych, Manos, Hoffstaedter, and Eickhoff (2019) for a recent review].

Data-driven modeling has gained traction at the macroscopic level with the rise of open-source brain imaging databases (such as OpenfMRI (Poldrack & Gorgolewski, 2017)). High-resolution structural and functional data from magnetic resonance imaging and diffusion tensor imaging (DTI) enable whole-brain modeling based on real anatomical features. Such type of models help to elucidate state transitions in brain activity and optimize external interventions to control brain states. The Virtual Brain (TVB) platform (Ritter, Schirner, McIntosh, & Jirsa, 2013; Sanz-Leon, Knock, Spiegler, & Jirsa, 2015; Sanz Leon et al., 2013), for instance, integrates functional MRI and DTI datasets to build individualized models, using coupled oscillators to represent regional activity. TVB has contributed significantly to the understanding of neurological disorders and serves as a testing ground for therapeutic interventions [see e.g., Courson, Quoy, Timofeeva, and Manos (2024); Monteverdi et al. (2023); Stefanovski et al. (2021) and references therein].

With advancements in computing resources and simulation technologies, integrating models across different scales has become both feasible and essential to strike a balance between retaining detailed information and achieving a broad-scale understanding. Recently, a co-simulation framework was introduced that employs NEST (a widely used simulator for point neurons and large neural networks) and TVB (broadly used and optimized for macroscale modeling of brain activity). This work has pioneered cross-scale modeling (Kusch et al., 2024) and has demonstrated the benefits of integrating models across spatial levels. Recently, Schirner et al. (2022) provided a comprehensive overview of similar software tools available within the European digital neuroscience platform, EBRAINS. A notable application of TVB-Multiscale is the virtual deep brain stimulation model (Meier et al., 2022; Shaheen, Pal, & Melnik, 2022), demonstrating its utility in multiscale simulations.

However, integrating microscopic and macroscopic models remains technically challenging, especially when one is interested in modeling morphological details and large-scale brain dynamics at the same time, possibly in addition to creation/deletion of synaptic elements associated with neural plasticity mechanisms. At the core lies the vast amount of information being processed, billions of cells with thousands of connections each, and the immense gap in timescales, from microseconds in ion channel dynamics to minutes for plastic changes of the connectome. The holistic understanding on how such neural biophysical processes affect the whole-brain dynamics, could be leveraged, among other applications, in optimized development of pharmacological treatments and the assessment of neuromodulation or surgical interventions. By employing different temporal and spatial resolutions for independent parts of the simulation, it is possible to reduce the computational requirements. We employ the Arbor simulator (Abi Akar et al., 2019) and more specifically its most recent next-generation version (Cumming et al., 2024). Designed for single-neuron and large-scale network simulations, Arbor leverages GPU resources to enhance computational speed and energy efficiency. A spike-based interface for co-simulation of biophysically detailed neuronal networks is provided.

In this study, we present a novel co-simulation framework integrating Arbor with TVB. In this first Arbor-TVB co-simulator implementation, our goal is twofold: first, to successfully establish efficient communication between the two simulators that respects their different operational time scales and, second, to provide a use case example of the combined neural activity. To this end, we use a mouse brain connectome—a graph of arbitrarily sized brain regions interconnected by weights and tract-length values—where each region represents the mean mass neural activity of a brain area modeled by a macroscopic model. Both the connectome matrices and the region models are provided by TVB. Then, using the co-simulation interface we replaced one TVB node with a detailed neural network with detailed neurons using Arbor. This neural population allows the user to choose an available model or easily develop a new one in Arbor and to define the details in the local connectivity of the population. We generate locally activity similar to epileptiform seizures in the network of detailed cells and monitor their propagation in the brain connectome with TVB. In this proof-of-concept study, we use a Hodgkin-Huxley-based model (Depannemaecker et al., 2022) placed on a single compartment which allows us to simulate different types of neural activity (e.g., spiking, bursting, seizure-like, etc.) by controlling a single parameter value.

## MATERIALS AND METHODS

### The Arbor simulator

Arbor is an open-source library for building simulations of biophysically detailed neuron models (Abi Akar et al., 2019). It provides an alternative to software like NEURON (Carnevale & Hines, 2006), but with a strong emphasis on modern hardware and scalability to large-scale systems (Hines, 1984). Its overall set of capabilities allows Arbor to model neural networks at a level of resolution beyond point models to explore phenomena like dendritic computation. Thus, support for bulk-synchronous parallelism via MPI, shared memory parallelism by utilizing a thread-pool and job system is central to Arbor, and certain cell types—primarily cable cells—can further leverage SIMD and GPU hardware. Arbor is written in *C*++, though most users interface with it through an intuitive, high-level Python interface built on top of the lower level implementation. The underlying numerical model of Arbor is the cable equation:

(1) $$c_{\text{m}}\frac{\partial V}{\partial t}=\frac{\partial }{\partial x}\left(\sigma \frac{\partial V}{\partial x}\right)+i_{\text{m}}$$

where the membrane potential V is computed over the morphological structure of the neural tree; the spatial coordinate x and derivative are to be understood within this structure (Hodgkin & Huxley, 1952a, 1952b, 1952c; Hodgkin, Huxley, & Katz, 1952; Loligo, 1952; Scott, 1975; Thompson & Kelvin, 1855; Von Helmholz, 1850). The parameters $c_{\text{m}}$ and σ define the membrane capacitance and longitudinal resistance. The trans-membrane current density $i_{\text{m}}$ models the entirety of ionic and non-ionic currents. In both NEURON and Arbor, these are calculated from user-specified sets of differential equations, potentially varying along the morphology. The equations for $i_{\text{m}}$ and V are solved in alternation (Lie-Trotter splitting) using a first-order implicit method.

### Single neuron and network models in Arbor

**Figure 1.**
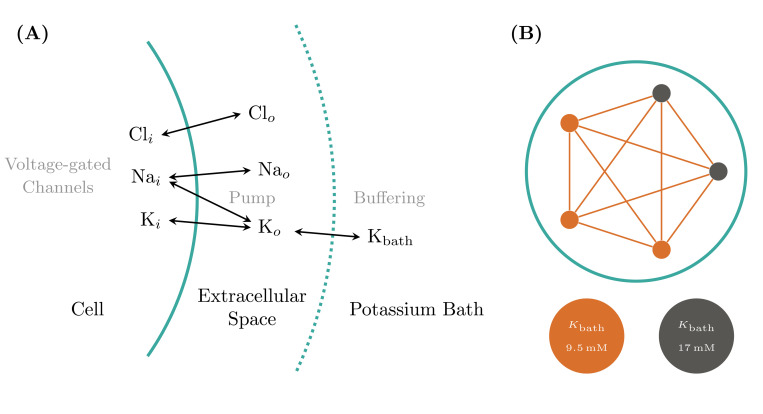
Biophysical neuron model and Arbor network. (**A**) For single cell dynamics three ion concentrations (K, Na, and Cl) are modeled in the cell’s interior and a thin shell of its extracellular medium. The latter is, in turn, surrounded by a bath of a fixed Potassium concentration ${\text{K}}_{\text{bath}}$. The model simulates changes to the concentration in addition to the current contributions based on three voltage-gated ion channels, an active pump between Potassium and Sodium, and the buffering effect of the surrounding Potassium bath. (**B**) We choose typical values of $K_{\text{bath}}$ for the single models to generate the tonic spiking (healthy) and seizure-like event (pathological) behaviors. In most cases, a fully connected network using exponential synapses with weight w=0.5 is used. As an example, we show here the network instantiation for N=5 and f=0.2.

**Figure 2.**
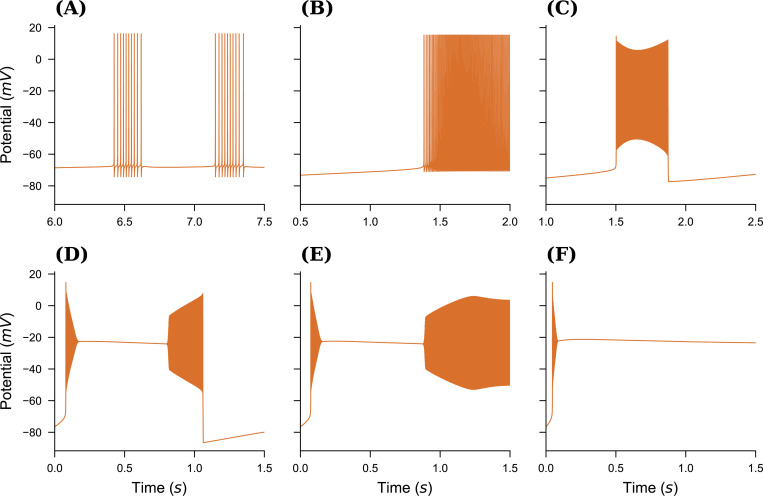
Different neural spiking patterns. (**A**) Spike Train, $K_{\text{bath}}=7.5\text{mM}$, (**B**) Tonic Spikes, $K_{\text{bath}}=9.5\text{mM}$, (**C**) Bursting, $K_{\text{bath}}=12.5\text{mM}$, (**D**) Seizure Like Event (SLE). $K_{\text{bath}}=17.0\text{mM}$, (**E**) Sustained Ictal Activity (SIA), $K_{\text{bath}}=17.5\text{mM}$, and (**F**) Deolarization Block, $K_{\text{bath}}=22.5\text{mM}$. Note that by setting $K_{\text{bath}}=4\text{mM}$, one can obtain Resting State (RS) activity too (result not shown here).

We begin by selecting a dynamical model that allows for relatively easy yet realistic simulation of a broad spectrum of neural activity at the single-neuron level, governed by a small set of biophysical parameters. The neural model from (Depannemaecker et al., 2022) was formulated for Arbor in the Neuron MODeling Language (NMODL). The following equations form the slow part of the system, describing the evolution of ion concentrations due to voltage-gated channels, active pumps, and buffering by an external bath see Figure 1(A) for a schematic of the dynamics. It describes the ionic exchanges between the intracellular and extracellular spaces (ICS, ECS) of a neuron immersed within an external bath, acting as a potassium buffer of concentration ${\text{K}}_{\text{bath}}$. Ions flow between the ICS and ECS through a sodium-potassium pump and the sodium, potassium and chloride voltage-gated channels, driving changes in the internal $\left({\text{K}}_{i},{\text{Na}}_{i},{\text{Cl}}_{i}\right)$ and external $\left({\text{K}}_{o},{\text{Na}}_{o},{\text{Cl}}_{o}\right)$ ionic concentrations. By gradually increasing the external bath concentration of potassium ions ${\text{K}}_{\text{bath}}$, the model sequentially presents these patterns: resting state (RS), spike train (ST), tonic spiking (TS), bursting, seizure-like events (SLE), sustained ictal activity (SIA) and depolarization block (DB), see Figure 2. The fast dynamics of the membrane potential V are modeled in Arbor via the cable equations, see above, which require computing the ion current densities $i_{\text{X}}$ used in the simulator update as:

$$i_{\text{X}}=g_{\text{X}}\left(V-E_{\text{X}}\right)\;E_{\text{X}}=C\cdot \text{log}\left(\frac{{\text{X}}_{o}}{{\text{X}}_{i}}\right)$$

with the ion species X={K,Na,Cl} and a non-ion current density:

$$i_{\text{pump}}=\frac{\rho }{\left(1+\text{exp}\left(10.5-0.5{\text{Na}}_{i}\right)\right)\left(1+\text{exp}\left(5.5-{\text{K}}_{o}\right)\right)}.$$

Following the Hodgkin-Huxley model, conductivities $g_{\text{X}}$ are written as:

$$g_{\text{K}}=g_{0,\text{K}}n+g_{l,\text{K}}\;g_{\text{Na}}=g_{0,\text{Na}}mh+g_{l,\text{Na}}\;g_{\text{Cl}}=g_{0,\text{Cl}}$$

The — internal i and external o — ion concentrations are modeled as:

$${\text{K}}_{i}={\text{K}}_{0,i}+\Delta {\text{K}}_{i}\;{\text{Na}}_{i}={\text{Na}}_{0,i}-\Delta {\text{K}}_{i}\;{\text{Cl}}_{i}={\text{Cl}}_{0,i}\;{\text{K}}_{o}={\text{K}}_{0,o}-\beta \Delta {\text{K}}_{i}+{\text{K}}_{g}\;{\text{Na}}_{o}={\text{Na}}_{0,o}+\beta \Delta {\text{K}}_{i}\;{\text{Cl}}_{o}={\text{Cl}}_{0,o}$$

The variables $\left\{\text{Δ}{\text{K}}_{i},{\text{K}}_{g}\right\}$ evolve as:

(2) $$\frac{d\text{Δ}{\text{K}}_{i}}{dt}=-\gamma \left(i_{\text{K}}-i_{\text{pump}}\right)$$

(3) $$\frac{d{\text{K}}_{g}}{dt}=\epsilon \left({\text{K}}_{\text{bath}}-{\text{K}}_{o}\right)$$

where γ converts current density $i_{\text{X}}$ to molar flux, summarizing the effect of the ion pump in Figure 1(A) and external buffer. Finally, fast dynamics were reduced and adjusted to mammalian neurons:

$$\begin{array}{lll} \frac{dn}{dt} & = & \frac{1}{\tau }\left(n-n_{\infty }(V)\right)\\ n_{\infty }(V) & = & \frac{1}{1+\text{exp}(-(19+V)/18)}\\ m=m_{\infty }(V) & = & \frac{1}{1+\text{exp}(-(2+V/12))}\\ h=h(n) & = & 1.1-\frac{1}{1+\text{exp}(3.2-0.8n)} \end{array}$$

based on the observations that the reaction of the Sodium gating variable to changes in V is nigh instantaneous and h(t)+n(t)=const. The resulting ion channel was added to a basic, spherical, single-compartment neuron. After implementing this biophysical model, we reproduced the firing patterns using the parameters of the reported model [Figure 2 (A–F)], see also Depannemaecker et al. (2022) for more details and motivation for model parameter choices. Note that despite the values given in the original publication, neither the Arbor nor the published reference implementation produces the depolarization block pattern at $K_{\text{bath}}=20\;\text{mM}$ but only at around $K_{\text{bath}}=22.5\;\text{mM}$. From here, a simple model network was developed, comprising N total cells, with a mixture of healthy f⋅N and pathological (1−f)⋅N cells, where both sub-populations are assigned individual values for $K_{\text{bath}}$, sketched in Fig 1. Cells are connected using exponential synapses with an internal weight of w=0.5 chosen to produce an activity similar to Rabuffo et al. (2024) which uses delta synapses.

### TVB network model

Following Deco et al. (2013), we used the reduced Wong-Wang model (Wang, 2002) to simulate resting-state activity and to investigate the dynamics of local brain regions embedded within a large-scale brain network. The mean firing rate $H\left(x_{I}\right)$ and mean synaptic gating variable $S_{I}$ of region I are described by:

(4) $$\frac{dS_{I}}{dt}=-\frac{S_{I}}{\tau_{s}}+\left(1-S_{I}\right)\gamma H\left(x_{I}\right)$$

(5) $$H\left(x_{I}\right)=\frac{ax_{I}-b}{1-\text{exp}\left(-d\left(ax_{I}-b\right)\right)}$$

(6) $$x_{I}=\omega J_{N}S_{I}+GJ_{N}\sum_{K}c_{IK}S_{K}+I_{0},$$

with ω=1

the local excitatory recurrence, $c_{IK}$ the strength of the structural connection from the local area I to K, and G a global coupling strength. The parameters are set to the same values used in the TVB implementation. $J_{N}=0.2609\;\text{nA}$ is the synaptic coupling of NMDA receptors and $I_{0}=0.33\;\text{nA}$ is the baseline external input. The kinetic parameters are $\tau_{s}=100\;\text{ms}$ and γ=0.641. The parameters of the input-output function H are $a=0.27\;{\text{nC}}^{-1}$, b=0.108kHz, and d=154ms. Depending on the tuning of G, the system exhibits a multi-stable regime, with steady states of high and low spiking activity. Here, we set G=0.096.

### Co-simulation framework of Arbor and TVB

**Figure 3.**
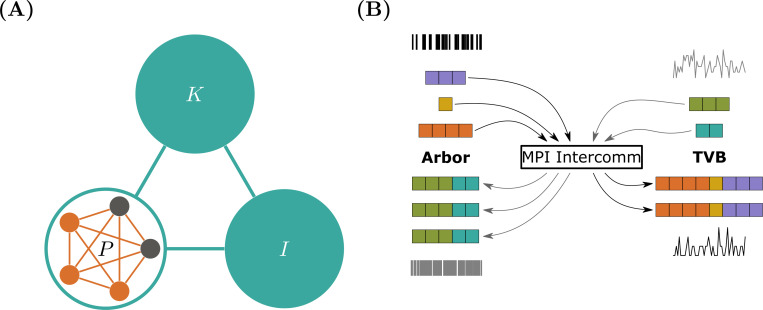
Arbor-TVB co-simulation schematic and communication pattern. (**A**) In a TVB simulation of regions I, K, and P; one region P will be replaced by a proxy containing a network of detailed cells simulated in Arbor. Regions are connected via the weights of the connectome and produce an activity values based on the chosen region model. When crossing the boundary between TVB and Arbor models, care needs to be taken to convert between discrete action potentials in Arbor to contiguous, region-model-specific variables in TVB. (**B**) Spikes generated by Arbor and TVB – converted from activity values interpreted as mean spiking rates – are exchanged using an MPI intercommunicator and the All-gather primitive. This is equivalent to concatenating all contributions from all Arbor MPI ranks and sending the result to all TVB ranks and vice-versa.

Both Arbor and TVB offer support for attaching a second simulator to perform co-simulation, potentially at different scales. Co-simulation from the TVB’s viewpoint is the simpler technology of the two frameworks, since TVB is designed to execute as a single process. TVB allows for exchange of any variable relevant to the region models and any number of variables. The co-simulation partner is encapsulated in one or more TVB regions, called proxy nodes, see Figure 3(A). These proxies present a conforming interface to TVB; exchanging the salient variables as a table, one row per time-step, one column per variable. As TVB advances in lockstep on a global time-step, this is almost identical to normal operation. However, co-simulation introduces the concept of an *‘epoch’* to TVB, i.e., the length of time that conforms to the smallest delay $\tau_{\text{min}}$ in the set of inter-region connections delays $\tau_{IJ}$. These delays are part of the connectome data used to construct a TVB simulation. (In the case that a connectome contains vanishing delays these must be replaced with a finite value. Further, it is required that the time-step evenly divides $\tau_{\text{min}}$.) Co-simulation thus can advance all nodes, including the proxy, for one epoch $\tau_{\text{min}}$ without exchanging data. This is correct as an event emanating from any region I at time t influences any other region J at time $t+\tau_{IJ}\geq t+\tau_{\text{min}}$. Only after an epoch, data need to be exchanged between the proxy and the rest of the TVB regions. A TVB–NEST demonstration has been published to showcase the interaction between a local network of spiking neurons and the whole-brain network dynamics (Kusch et al., 2024).

Arbor has a fundamentally different design in terms of connectivity. Interaction between physically separate cells is mediated by action potentials, which are triggered by dedicated sources, e.g., when the membrane potential crosses a configurable threshold. Cells are connected by wiring these sources to corresponding sinks like synapses via an abstract connection object comprising a delay and weight, modeling transmission and attenuation via an axon. In contrast to TVB, Arbor is fundamentally a distributed system and internally employs the same approach to decoupling via the minimum network delay as explained above. To initiate co-simulation, Arbor exposes an additional interface comprising the external connections, i.e., those terminating at cells managed by Arbor, but originating outside, and functionality to exchange spikes with Arbor. On the technical side, the latter part leverages MPI_Allgatherv through an inter-communicator and effects that the concatenation of all spikes sent from all MPI ranks running TVB arrive on all ranks running Arbor and vice versa [Figure 3(B)]. This allows co-simulation in conjunction with arbitrary numbers of ranks on both sides and even in compounds with more than two simulators.

Finally, bi-directional translation between TVB’s variable concept and Arbor’s representation of action potentials is required. As the former depends on the region models used, we chose to bundle this with the remaining TVB functionality as part of the Arbor proxy node. For the TVB models used in this study, the main variable is the per-region mean activity rate $\nu_{I}$ which is conceptually compatible with the concept of spike generation. For each region I connected to the proxy node P, i.e., with connectome weight $c_{IP}>0$, a set of synthetic events needs to be generated such that the mean activity conforms to $\nu_{I}$. This is an ambiguous process, even if we prescribe a population (list of cell identifiers) and a per-cell distribution, e.g., a Poisson point process, from which to draw events, which likely must be resolved by ensembles of simulations. In general, this is both model dependent and mathematically intractable, so we leave the general case as a customization point in the framework. For our running example, however, we make the following choice: Event timings for the current step k will be drawn from a uniform distribution and dispatched to all cells in the Arbor network, Note that while these events are created at given time a per-connection delay is applied and thus delivery occurs at a later time.

**Figure 4.**
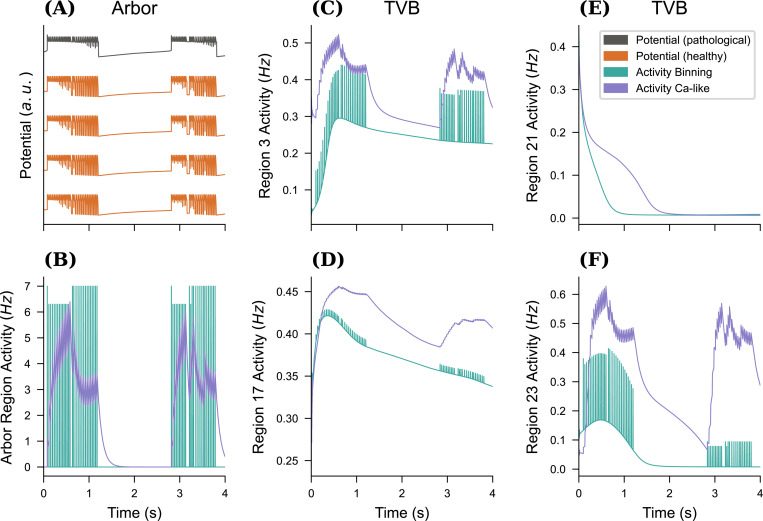
Impact of conversion method on activity exchange. For an all-to-all connected network of a mixture of 10 pathological cells $\left(K_{\text{bath}}=17.5\text{mM}\right)$ and 90 healthy $\left(K_{\text{bath}}=9.5\text{mM}\right)$ neurons we plot the membrane potential traces (**A**) for four healthy and one pathological cell in (**A**). This simulation is repeated for two choices of generating the activity of the detailed network, either spikes were binned into buckets of width Δt to extract instantaneous rates, or the differential equation 7 emulating the change in Calcium concentration of a biological cell after spiking was used (with τ=100ms and $\beta =\frac{0.1}{N}$). The resulting activity traces for the Arbor (**B**) network and selected TVB nodes ((**C**)–(**F**), out of 98 regions).

The inverse direction, converting spike events to mean rates, while being well-defined, is still subject to customization. We explore two options here. First, simple running averages, i.e. all spike originating within the Arbor network during the current epoch are collected and sorted into bins of width Δt. This list is then normalized to the cell count and time step and sent to TVB as the mean activities as a function of time. Although straightforward, this can lead to unrealistically rough activity traces, especially if cell populations are small. Second, as inspired by high-speed calcium imaging experiments (Grewe, Langer, Kasper, Kampa, & Helmchen, 2010), a mechanism of tracking cell activity via calcium level is implemented as:

(7) $$\frac{dC_{p}}{dt}=-\frac{C_{p}(t)}{\tau }+\beta \sum_{t_{\text{spike},p}}\delta \left(t-t_{\text{spike},p}\right),\;C_{p}(0)=0$$

with per cell p with decay parameter τ and weight β. Computing the activity becomes the average:

(8) $$\nu_{P}(t)={\langle C_{p}(t)\rangle }_{p\in P},$$

yielding a smooth trace. This method of converting discrete spiking events into a continuous interval variable is also used in a few plasticity models recruiting a negative feedback control mechanism such as synaptic scaling (Van Rossum, Bi, & Turrigiano, 2000) and homeostatic structural plasticity (Butz & Van Ooyen, 2013; Diaz-Pier et al., 2016; Lu et al., 2024) models. Figure 4 compares the impact of this choice on the macro-scale network. In small networks and over short timescales defined by the epoch length as shown in the example, spiking activity occur in noncontiguous bursts, which is dubious in conjunction with the smooth dynamics of the chosen TVB model. Figure 4(A) shows the propagation of this noncontiguous activity into the TVB regions, while using the Ca-like model (B) provides smooth dynamics in both the Arbor and TVB models. We thus will use the latter in all simulations from here on out. In general, both methods require scaling by the number of cells in the proxy region to arrive at a scale-free activity measure. A local scaling factor $G_{\text{A}}$ is used to convert between the activity detailed network and the region model specific TVB activity measure. In general, $G_{\text{A}}$ needs to be adjusted to the choice of connectome and TVB model, similar to the choice of the global coupling strength G in the RWW model, here $G_{\text{A}}=100$ proved to produce acceptable resuls.

## RESULTS

So far, we have described the components of the co-simulation framework, that is the model of individual detailed cells, the prototypical network used for detailed cells, and the neural mass model used in TVB, and the manner of establishing a bi-directional connection between Arbor and TVB, as well as the methods of converting between spikes and rates. In summary, an Arbor–TVB co-simulation model consists of the following key components:

- A TVB model based on the connectome and node dynamics.
- One or more internally connected network models in Arbor.
- A specified set of TVB nodes where the Arbor models will be placed.
- A defined mechanism for routing events from TVB to individual cells in Arbor.
- A method for translating Arbor-generated events into TVB variables.
- A translation process for converting TVB variables into events originating from synthetic cells.

Each of these components serves as a customization point for the user. While reasonable default configurations can be provided for some, others require user-defined specifications to suit specific modeling needs.

The single cell model has been demonstrated to exhibit the necessary range of healthy and pathological behaviors. We have also motivated our choice for converting spikes to rates of using a biologically-inspired exponential smoothing filter via a Ca-like activity over simple binning and fix parameters to τ=100ms and $\beta =\frac{0.1}{N}$. This normalization is important as it yields results that invariant under changes in the number of cell N in the detailed network.

### Propagation of pathological states in a network of detailed cells

**Figure 5.**
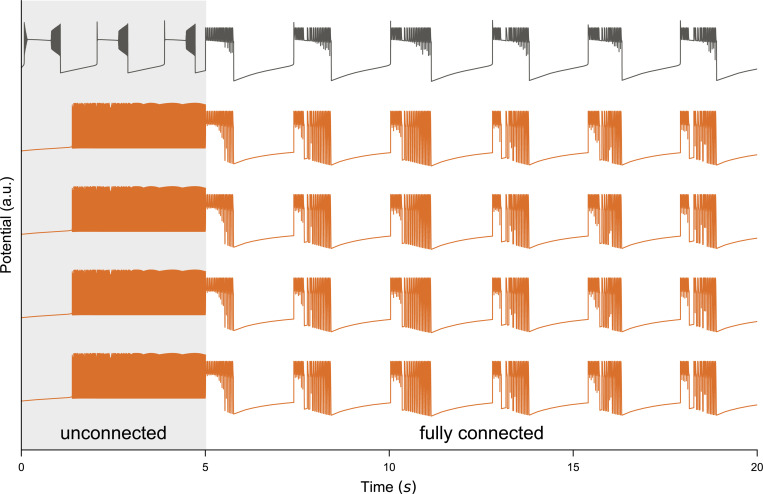
Effect of pathological patterns on the network. A network simulation of detailed cells comprising 80% healthy cells ($K_{\text{bath}}=9.5\text{mM}$ tonic spiking) and 20% pathological cells ($K_{\text{bath}}=17.0\text{mM}$, SLE). Shown are membrane potentials for each cell. The simulation was integrated for 5s (shaded area) without internal connections during which cells follow their individual patterns as in Fig 2. After this initial period the network was switched to a fully connected graph and settles into a new equilibrium state driven by the single pathological cell.

As a first step, we consider a network of detailed cells without embedding into a co-simulation and study the impact of a small population of pathological cells on the network dynamics. For this, a simulation of 80% healthy cells ($K_{\text{bath}}=9.5\;\text{mM}$, tonic spiking) and 20% pathological cells ($K_{\text{bath}}=17.0\;\text{mM}$, SLE) was set up without any internal connectivity. This simulation was run forward in time for T=5s and the resulting membrane potentials are shown in Figure 5. During this phase, all cells follow their inherent behavior independently as in Figure 2. After this initial undisturbed simulation, integration was halted, the network was transitioned to fully connected, and simulation resumed. The effect of this switch on all cells is immediate and the network now follows the general pace of the pathological neurons modulated by tonic spiking, see Figure 5, T≥5s.

### Seizure detection and propagation patterns

**Figure 6.**
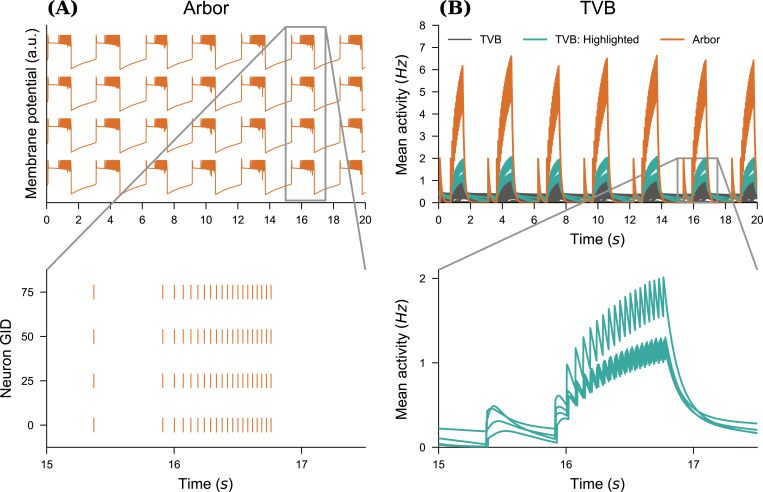
Multiscale seizure propagation. (**A**) Membrane potential and raster plot for four neurons in the detailed fully-connected Arbor neural network. Note that the membrane potential time-series are similar across all neurons. (**B**) Mean firing rate of various TVB local brain areas versus the Arbor activity. We track a seizure after a transient period, so that all brain areas have reached their baseline activity. The zoomed-in sections show the propagation of the seizure. We highlight and show detail the four timeseries with the largest deviation in activity.

To induce seizure propagation in mice brain, we use the structural connectivity derived from the Allen mouse brain atlas (Oh et al., 2014), also used in Melozzi, Woodman, Jirsa, and Bernard (2017), to embed the TVB nodes. Following the work presented in Courson et al. (2024), the proxy node modeling the Arbor population of healthy and diseased neurons is set within left Hippocampus, i.e., either left-field CA1 (l CA1), left-field (l CA3), or left Dentate Gyrus (l DG). The proxy node consists in a fully connected network of N neurons. The connections are facilitated using exponential synapses whose weight is tuned to produce sustained network activity. All neurons produce SLE with $K_{\text{bath}}=17.0\;\text{mM}$. The activity of the spiking neural network is smoothed following Eq. 7, with τ=100ms. Figure 6 (A) shows the evolution of the membrane potential for the individual neurons in the Arbor network, all neuron exhibiting the same dynamics. As discussed earlier, the initial shape of the SLE is altered by the coupling of the neurons. The neurons are synchronously recruited into seizure patterns of activity. The threshold used for spikes detections was tuned to −25mV to capture the seizure dynamics. We run simulations over 20s s, and investigate the effect of SLE emergence in the diseased network once all brain areas have reached their steady state. The production of SLE patterns in the diseased Arbor node generates changes in the mean firing rates of TVB local brain areas. Even though changes in the activity occur in most of the brain areas, these fluctuations are generally small, therefore we use region-specific thresholds to detect the seizures. In this section, we characterize a seizure as an SLE activity pattern causing the mean activity to increase at least by 55% of its baseline resting value. A seizure is then detected at the first peak of increased firing activity.

In Figure 6(B), we show the time-series of some TVB nodes’ mean firing rate over the entire simulation, here with N=100 in the detailed network. A large transient period is necessary before all nodes reach their steady-state. The emergence of recurrent SLE patterns in the Arbor node triggers SLE patterns in the dynamics of the TVB nodes. In the zoomed-in sections, we show a seizure in more detail. The red trajectories highlight the brain areas where the seizure is detected. Note that even non-recruited areas leave their steady-state, but the activity remains relatively weak for a seizure to be detected with this criterion.

**Figure 7.**
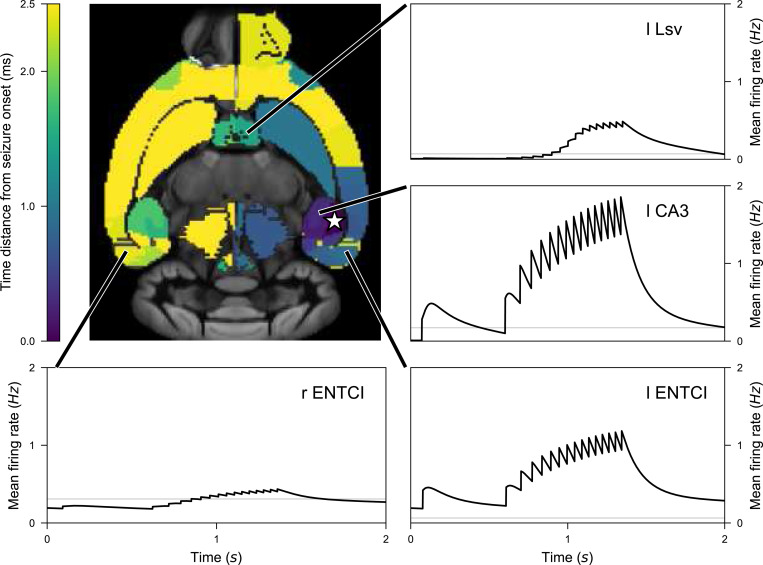
Propagation or a seizure originating in left-field l CA3 area of the mouse brain model. Time distance between seizure emergence in l CA3 (star marker) and spiking rate increase in each brain area. Time series of selected brain areas are displayed, starting at the detected onset of the seizure. For comparison we also show the baseline activities for these node, the activity in the absence of the Arbor node.

In Figure 7, we present the propagation of SLE originating in l CA1 (star marker), represented as a fully connected network of N=10000 neurons. Colors on the brain template show the time distance between seizure emergence in the diseased area and seizure arrival in the different brain regions. Following the detection procedure described above within 25ms a SLE is detected in brain areas inside and outside the hippocampal formations (HFs). Furthermore, we show the activity time-series of four healthy nodes of the brain network being recruited in the seizure, namely the ventral part of left Lateral Septal Nucleus (l LSv), l CA3, l ENTCl and r ENTCl. Note that both the baseline activity and the mean activity reached during the seizure differ depending on the brain area. In particular, since the Allen Mouse Brain SC has symmetrical connections between left and right hemispheres, l ENTCl and r ENTCl have the same baseline activity. However, as the SLE pattern emerges in left Hippocampus, l ENTCl reaches higher spiking rates.

### Performance of the Arbor-TVB co-Simulation Framework

Next, we briefly analyzed the performance of the running example on a single Apple M1 (2021) laptop. Arbor was built with support for MPI and SIMD (Arm Neon/SVE), and cells were bundled into groups of ten to leverage SIMD units. The overall runtime consists of four main components:

- Arbor model update,
- conversion from spikes to rates,
- TVB model update,
- conversion from rates to spikes.

**Figure 8.**
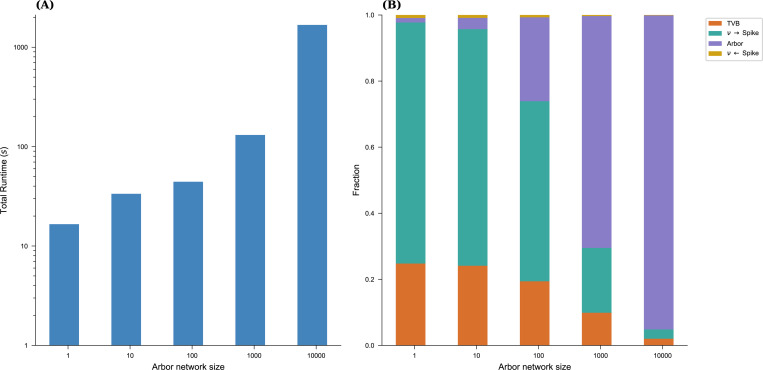
Performance of the running example. We graph the overall runtime (**A**) of the full co-simulation over the number of cells in the Arbor network as well as the fraction of the main contributions (**B**): time spent in both simulation engines and in converting between rates and spikes. TVB and rate to spike conversion are the most relevant cost center in the limit of vanishing Arbor network sizes while at large sizes, Arbor compute time dominates.

The Arbor update runs in parallel with the conversions between rates and spikes, as well as the TVB update. During spike exchange, both simulations synchronize, meaning the slower part must wait in the MPI collective, which accounts for the primary time spent in the collective call. Figure 8 illustrates the total runtime of a 10s simulation for the entire model described above, along with the relative contributions, for system sizes ranging from one to 10000 cells. Notably, at 10000 cells, nearly all computational time is spent within the Arbor network model. In future experiments, we plan to leverage additional hardware, including GPUs, to accelerate the Arbor side of the simulation. At this scale, TVB and the spike/rate conversions are potential bottlenecks that will require optimization, potentially through TVB’s JIT compilation and GPU acceleration. Additionally, further parallelization and porting of the conversion steps to a more performant programming environment remain promising avenues for improvement.

## DISCUSSION

In this work, we presented a co-simulation framework that offers a novel approach to bridging the gap between microscopic (spiking neuron) and macroscopic (mean-field) models. This framework integrates Arbor and TVB within a parallelized MPI environment, enabling a detailed yet computationally feasible representation of neural dynamics across scales. To model large-scale brain network dynamics, we used the mean-field reduced Wong-Wang model to reproduce resting-state dynamics. Simultaneously, a detailed spiking neural network was simulated with Arbor, employing a physiological model of seizures at the neuron level (Figure 2). The spiking activity of the population was then converted into a smooth trace for the proxy node, which was communicated with TVB [Figure 3(B)]. This co-simulation approach successfully captured the interplay between spiking activity and large-scale brain dynamics, where local neuron dynamics generate global activity wave-fronts. At the microscopic scale, we demonstrated that the structure of the detailed neural network influences its activity patterns, thereby affecting the shape of the activity wavefront (Figure 4).

As a proof of concept, we simulated the emergence of seizure-like activity patterns (SLE) in the mouse hippocampus, using the Allen Mouse Brain Structural Connectivity data. We modeled the diseased brain area with a small, fully-connected network of neurons SLE, which produces scale-free activity patterns. This approach offers a well-understood and easily controlled model, which is essential for demonstrating the usability of the technical substrate. However, it leaves significant potential for more complex cell models untapped. We would like to stress that this is not a limitation of the framework or its components, and it is possible build upon the model used here in future works.

Arbor has embarked on many types of computational studies as a new-generation simulator. It enables seamless conversion and simulation of single-neuron models from the NEURON simulator and supports simulations of both individual neurons and large-scale networks. Arbor accommodates various plasticity models, including spike-timing dependent plasticity, calcium-based synaptic tagging and capture, and structural plasticity. It has been used to study synaptic tagging and capture via the built-in diffusion unctionality [Luboeinski et al. (2024), under review]. Recent developments focus on co-simulation with membrane dynamics and external kernels, enabling dynamic connectivity modifications in a distance-dependent manner. With its high flexibility and scalability, Arbor stands out as a promising platform developed within the EBRAINS initiative to advance cross-scale simulations in computational neuroscience. Arbor is available as part of EBRAINS software distribution (ESD) on connected HPC centres and the EBRAINS collab via Jupyter Lab. Providing a bridge between morphologically detailed neurons and neural mass models encompassing the full brain spans a gap of scale from sub-micrometer to decimeter. It allows for placing the resolution — using Arbor and detailed models — where needed and using realistic, data driven environments everywhere else via TVB.

Despite its successes, the Arbor-TVB framework has some limitations. The co-simulation requires careful exploration and calibration of coupling parameters to ensure meaningful interactions between Arbor and TVB, which remains a challenge when generalizing to diverse neural models. Specifically, computational costs for large neural networks may necessitate further optimization in model parallelization and data handling. A near-term goal would be to incorporate new features, such as synaptic plasticity, which could offer valuable insights into how brain networks adapt and reorganize in response to disrupted activity.

From an epilepsy-seizure perspective, while the framework provides insights into seizure propagation, additional validation against empirical data would enhance its applicability to clinical settings. This co-simulation framework could enable a detailed investigation of the physiological sources of seizures. Understanding the impact of the structure of the diseased area on seizure patterns and propagation would be of great interest [see e.g., Garcia-Ramos et al. (2016); Netoff, Clewley, Arno, Keck, and White (2004)]. Specifically, we expect the inhibition and excitation ratios in the detailed neural network to play a critical role in seizure patterns [see e.g., Engel (1996); Liu, Grigorovsky, and Bardakjian (2020)].

## TECHNICAL TERMS

Arbor: A multi-compartment neuron simulation library; compatible with next-generation accelerators
The Virtual Brain (TVB): An open-source platform for constructing and simulating personalised brain network models
Co-simulation: Simulating a set of co-dependent phenomena using separate software packages, each providing input and/or output to each other over an interface
MPI (Message Passing Interface): A software library for sharing information across nodes distributed parallel programming, fundamental to larger computing clusters
Collective/Allgather: MPI primitive executed by all nodes simultaneously, synchronizing execution. Allgather will concatenate all nodes’ contributions and provide the output to all nodes
SIMD (Single Instruction Multiple Data): Lowest level of parallelism in a CPU, where a single instruction operates on multiple numbers simultaneously
GPU (Graphics Processing Unit): Originally used to render games, these highly parallel accelerators are now responsible for most of the processing power of supercomputers
JIT (Just In Time): Compile code to a more efficient format just before or even during execution
NMODL (Neuron MODeling Language): Domain-specific language used by Neuron and Arbor to describe the dynamics of ion channels and synapses using differential equations
